# Multimorbidity at time of death among persons with type 2 diabetes: a population-based study in Ontario, Canada

**DOI:** 10.1186/s12902-023-01362-x

**Published:** 2023-06-02

**Authors:** Laura C. Rosella, Ednah Negatu, Kathy Kornas, Casey Chu, Limei Zhou, Emmalin Buajitti

**Affiliations:** 1 Dalla Lana School of Public Health, University of Toronto, Health Sciences Building, 6th floor, 155 College Street, Toronto, ON M5T 3M7 Canada; 2 ICES, Toronto, ON Canada; 3 Institute for Better Health, Trillium Health Partners, Mississauga, ON Canada; 4 Temerty Faculty of Medicine, Toronto, Canada

**Keywords:** Multimorbidity, Mortality, Chronic conditions, Socioeconomic status, Immigrants

## Abstract

**Objective:**

Individuals with Type 2 Diabetes are likely to experience multimorbidity and accumulate multiple chronic conditions over their life. We aimed to identify causes of death and chronic conditions at the time of death in a population-based cohort, and to analyze variations in the presence of diabetes at the time of death overall and across income and immigrant status.

**Research design and methods:**

We conducted a retrospective cohort study of 2,199,801 adult deaths from 1992 to 2017 in Ontario, Canada. We calculated the proportion of decedents with chronic conditions at time of death and causes of death. The risk of diabetes at the time of death was modeled across sociodemographic variables with a log binomial regression adjusting for sex, age, immigrant status, area-level income. comorbiditiesand time.

**Results:**

The leading causes of death in the cohort were cardiovascular and cancer. Decedents with diabetes had a higher prevalence of most chronic conditions than decedents without diabetes, including hypertension, osteo and other arthritis, chronic coronary syndrome, mood disorder, and congestive heart failure. The risk of diabetes at the time of death was 19% higher in immigrants (95%CI 1.18–1.20) and 15% higher in refugees (95%CI 1.12–1.18) compared to long-term residents, and 19% higher in the lowest income quintile (95%CI 1.18–1.20) relative to the highest income quintile, after adjusting for other covariates.

**Conclusions:**

Individuals with diabetes have a greater multimorbidity burden at the time of death, underscoring the importance of multiple chronic disease management among those living with diabetes and further considerations of the social determinants of health.

**Supplementary Information:**

The online version contains supplementary material available at 10.1186/s12902-023-01362-x.

Type 2 diabetes is one of the most common chronic diseases in the world. In 2015, it was estimated that 415 million people aged 20–79 had diabetes, reaching 463 million in 2019 [1]. Most people with type 2 diabetes have more than one chronic condition, known as multimorbidity [2], which increases complexity of care and resource coordination [3, 4]. In particular, individuals with type 2 diabetes have a higher likelihood of experiencing cardiovascular diseases than the general population and are at risk of developing complications, such as stroke, renal disease, and ischaemic heart disease [5–7]. A range of other conditions have also been shown to be prevalent among those with diabetes, including hypertension, metabolic syndrome, and depression [8].

Historically, cardiovascular disease complications were the most common cause of death for those with type 2 diabetes [9]. In addition, diabetes has been found associated with premature death from cancers, infectious diseases, external causes, and degenerative disorders [9]. Multimorbidity, however, makes it difficult to characterize cause of death using standard approaches such as death certificates, meaning that as multimorbidity increases, mortality outcomes in those with diabetes become increasingly imprecise. Thus, understanding both how chronic conditions accumulate over the life course and causes of death among those with diabetes can signal opportunities for health system improvement, particularly where effective interventions exist [10, 11].

Variations in mortality among population sub-groups are difficult to quantify since this information is not typically collected in clinical or in vital statistics databases. Immigrants, for instance, have generally been shown to have better health outcomes than non-immigrants [12]. In Ontario, Canada, where almost 30% of the total population is comprised of immigrants [13], the all-cause mortality rate was almost 60% lower in immigrants compared to their Canadian-born counterparts from 2002–2012 [14]. However, less is known among those who have been diagnosed with diabetes, given that immigrants, particularly those with South Asian and African ethnicities, have an increased risk of developing diabetes [15]. Variations in mortality among those with diabetes across groups by socioeconomic status is also important to consider. In a study linking population health survey data to mortality files in the U.S., individuals in the lowest education and family income class were at more than two times increased risk of diabetes related deaths than individuals in the highest classes [16].

In this population-based study we used multiple linked administrative, vital statistics, and census data sources to identify chronic conditions present at the time of death, including diabetes, and analyzed variations in the presence of diabetes at the time of death overall and across socioeconomic status and immigrant status.

## Research design and methods

### Study population and design

We conducted a retrospective population-based analysis using all recorded deaths dated between January 1, 1992 and December 31, 2017. Eligible patients were residents of the province of Ontario, Canada, aged between 20 and 120 years old at date of death. We excluded those patients with an invalid health card, missing age or sex, or when the death was recorded before the diabetes diagnosis date. Datasets were linked using unique encoded identifiers and analyzed at ICES in Toronto, Ontario. ICES is an independent, non-profit research institute whose legal status under Ontario’s health information privacy law allows it to collect and analyze health care and demographic data, without consent, for health system evaluation and improvement. This study was approved by the Research Ethics Boards at the University of Toronto and Sunnybrook Health Sciences Centre.

### Data sources

The death information, including date of death and the corresponding cause of death, was captured from the Office of the Registrar General- Deaths (ORGD) file. Cause of death was coded using the International Classification of Disease 9^th^ and 10^th^ revision, with Canadian Enhancements (ICD-9/10-CA). Specifically, cause of death was based on the underlying cause of death indicated on decedents’ death certificates. The ORG-D was linked probabilistically to the Registered Persons Database (RPDB) with an overall linkage rate of 96.5% [17]. We also used the RPDB to identify death dates that were not captured in the ORG-D. The RPDB is a central population registry file that contains basic demographic information, such as birth date, sex, and postal code, for those who have ever received an Ontario health card number for the province’s universal health care system. The RPDB permits linkage with data holdings held at ICES.

The Ontario Diabetes Database (ODD) was used to identify decedents with a history of a diabetes diagnosis. The ODD is described in detail elsewhere [18]. Briefly, the ODD uses a validated algorithm applied to inpatient hospitalization records, same day surgery records, and physician billing claims data to determine the diagnosis of incident cases of diabetes in Ontario. The ODD has demonstrated 90% sensitivity and 97% specificity [18]. The definition for diabetes is 2 physician billing claims with a diagnosis for diabetes (diagnosis code: 250) or 1 inpatient hospitalization or same day surgery record with a diagnosis for diabetes (ICD-9 diagnosis code: 250; ICD-10 diagnosis codes: E10, E11, E13, E14; in any diagnostic code space) within a 2 year period. Physician claims and hospitalizations with a diagnosis of diabetes occurring within 120 days prior to and 180 days after a gestational hospitalization record are excluded.

Other administrative databases linked to identify history of chronic conditions prior to death included: the Canadian Institute for Health Information for hospital admissions and day surgery (DAD/SDS); the Ontario Mental Health Reporting System for inpatient care related to mental health; the National Ambulatory Care Reporting System for all records of emergency room visits; and the Ontario Health Insurance Plan claim database for outpatient physician visits. We utilized several disease-specific registries that contain regularly updated population cohorts and were created by applying validated data algorithms to inpatient and outpatient (office visit) data, specifically: Ontario Asthma Database [19], Ontario Cancer Registry [20], Ontario Congestive Heart Failure Database [21], Ontario Chronic Obstructive Pulmonary Disease Database [22], Ontario Rheumatoid Arthritis Database [23], Ontario Hypertension Database [24], and Ontario Crohn’s and Colitis Cohort Database [25]. The look-back period included all available data before death.

The Ontario portion of the Immigration, Refugees, and Citizenship Canada (IRCC) Permanent Resident Database was used to identify immigrants in our study population. These data contain records for over 3 million individuals at the time of landing in Ontario from January 1985 to December 2012, and was linked to the RPDB with a 86.4% overall linkage rate [17]. Neighbourhood-level measures of income were calculated using data from the 2001, 2006, and 2016 Canadian census.

### Exposures

Comorbidity burden was captured in the form of total Aggregated Diagnosis Groups (ADG) scores which were derived from diagnosis codes recorded in both ambulatory and inpatient care in a period of 2 years prior to death. Individual diseases or conditions are placed into a single ADG based on five clinical dimensions: duration of the condition (acute, recurrent, or chronic); severity of the condition (e.g., minor and stable versus major and unstable); diagnostic certainty (symptoms focusing on diagnostic evaluation versus documented disease focusing on treatment services); etiology of the condition (infectious, injury or other); and specialty care involvement (medical, surgery, obstetric, hematology, etc.). Individuals are assigned up to 32 ADGs, with higher ADG scores reflecting a greater burden of illness. ADG scores were calculated using The Johns Hopkin’s ACG^**®**^ System Version 10.0.1 [26].

Area-level income information was obtained from the census and applied to individual decedents according to the dissemination area (DA), which represents the smallest geographic census area in which the individual resided. Individuals were assigned to a DA based on their postal code at time of death. Because area-level income information from the census is only updated once every 5 years, we used a nearest-census approach for linking area-level income to individual decedents. Specifically, we used 2001 income information for deaths between 1992 and 2003, 2006 income information for deaths between 2004 and 2010; and 2016 census data for deaths between 2011 and 2017. The long-form census was not mandatory in Canada in 2011, so 2011 data are considered non-representative and were not used in this study. After linking to area-level income, individuals were categorized into income quintiles ranging from 1 (20% lowest income) to 5 (20% highest income).

Immigrant status was defined as landed immigrants or refugees who were not Canadian citizens by birth and arrived in Ontario between 1985 and 2012.

### Outcomes

The primary outcome of interest was a diagnosis of diabetes prior to death, which was ascertained using all years of available data prior to death. Other lifetime chronic conditions were examined in the same manner and included: acute myocardial infarction, asthma, cancer, cardiac arrhythmia, chronic coronary syndrome, chronic obstructive pulmonary disorder, congestive heart failure, Crohn’s or colitis disease, dementia, hypertension, mood disorder, other mental health disorders, osteo- and other arthritis, osteoporosis, rheumatoid arthritis, renal failure, and stroke. Diagnostic codes and details are located in the Supplemental Table S1. Multimorbidity was defined as the co-occurrence of two or more of these chronic conditions at the time of death.

We also examined all-cause mortality, premature mortality, amenable mortality, and causes of death among individuals who died with and without a diabetes diagnosis. Premature mortality was defined as a death before 75 years of age, from any cause. This age cut-off was used to be consistent with other work in Canada, and reflects deaths that may have been avoidable given appropriate medical or population health management. Established classification lists were used to identify premature deaths from specific causes that are considered amenable to the health system [27–29] (amenable mortality – see Supplemental Table S2 for complete list). Cause of death was assigned using the ICES-derived cause of death variable in the ORG-D, which is based on Medical Certificate of Death coding of the underlying cause of death, enhanced via linkages with other provincial data holdings, and converted to ICD-9 codes. Cause of death groupings were based on chapters of ICD-9: diseases of the cardiovascular and circulatory system (ICD-9 codes 390–459), cancers (ICD-9 codes 140–239), external causes of injury and poisoning (ICD-9 codes 800–999), diseases of the respiratory system (ICD-9 codes 460–519), endocrine, nutritional and metabolism diseases (ICD-9 code 240–279), and infectious diseases (ICD-9 codes 001–139). All other deaths were assigned the cause of death category of ‘other’.

### Statistical analysis

We summarized baseline characteristics of all deaths, stratifying based on diabetes status. Comparisons were carried out by Pearson’s chi square test for categorical variables and ANOVA or Kruskal–Wallis test for continuous variables.

We modeled the relative risk of diabetes diagnosis prior to death using log-binomial regression. The primary outcome of these models was diabetes diagnosis, resulting in relative risk estimates for each covariate, based on their associations with diabetes prevalence among Ontario decedents.

The following covariates were added into the multivariable models sequentially for adjustment: sex, age at time of death, calendar year, immigrant status, income quintile, and ADG score. Age was included as a continuous variable. Because ADG score is a summary measure of health status and comorbidity burden, we did not include individual chronic conditions or multimorbidity in the models.

All analyses were performed using SAS version 9.4 (SAS Institute, Cary, NC) in a UNIX environment.

## Results

The study population consisted of all deaths registered in the province of Ontario between January 1, 1992 and December 31, 2017. After excluding individuals aged < 20 or ≥ 120 years (*n* = 42 334), non-Ontario residents (*n* = 5 443), and those with a death date prior to the diabetes diagnosis date (*n* = 46), a final cohort of 2 199 801 decedents remained, 646 841 of which had diabetes. The cohort flowchart for this study can be found in Supplemental Fig. S3.

### Cohort characteristics

Table 1 displays the characteristics of the total cohort by diabetes status. On average, those that died with diabetes were approximately 2 years older, had higher comorbidity scores, and were more likely to be males, immigrants, and in the lowest income quintile than the sub-set that died without diabetes. The majority of the deaths cohort experienced multimorbidity, with 99.1% of decedents with diabetes and 87.1% of decedents without diabetes having 2 or more conditions at the time of death. Over half of the cohort died with 5 or more conditions, but this was more prevalent among those that died with diabetes than without diabetes (77.4% compared to 43.2%). Premature deaths were more prevalent among those that died without diabetes (40.2%) compared to with diabetes (37.4%). In contrast, amenable deaths were more prevalent among those with a diabetes diagnosis (20.9%) compared to without diabetes (18.3%).

**Table 1:** Characteristics of all adult decedents at time of death, by presence of diabetes, Ontario, Canada, 1992–2017

**Characteristic**	**Overall** ***N* = 2 199 801**	**Presence of diabetes at time of death**	
		**Yes** ***N* = 646 841**	**No** ***N* = 1 552 960**
**Age** (years) (mean (SD))	75.5 (14.8)	76.8 (11.9)	75.0 (15.9)
(median (IQR))	78 (68–86)	78 (70–85)	79 (66–87)
**Age**			
18–34	1.7	0.3	2.3
35–64	18.6	14.7	20.3
65–79	32.7	38.7	30.1
80+	47.0	46.3	47.3
**Sex**			
Males	50.5	53.5	49.3
Females	49.5	46.5	50.7
**Immigrant status**			
Immigrant	3.6	4.4	3.2
Refugee	0.5	0.6	0.5
Long-term resident	95.9	95.0	96.3
**Area Income Quintile**			
Quintile 1 (lowest)	24.0	25.7	23.3
Quintile 2	21.4	22.1	21.1
Quintile 3	19.3	19.3	19.2
Quintile 4	17.4	16.9	17.6
Quintile 5 (highest)	16.9	15.2	17.6
Missing	1.0	0.8	1.1
**ADG score**^**a**^ (mean (SD))	11.2 (4.4)	12.3 (4.1)	10.7 (4.4)
(median (IQR))	12 (8–14)	13 (10–15)	11 (8–14)
**Degree of multimorbidity**			
0 conditions	2.8	0.0	3.9
1 condition	6.6	1.0	9.0
2 conditions	10.5	3.5	13.4
3 conditions	12.9	7.2	15.3
4 conditions	13.9	11.0	15.2
5+ conditions	53.3	77.4	43.2
**Premature deaths**	39.4	37.4	40.2
**Amenable deaths**	19.1	20.9	18.3

*SD* Standard deviation, *IQR* Interquartile range

^a^ADG refers to Aggregated Diagnosis Groups scores which captures comorbidity burden calculated from patient diagnostic codes for ambulatory and inpatient care

### Distribution of chronic conditions at time of death

**Fig. 1:**
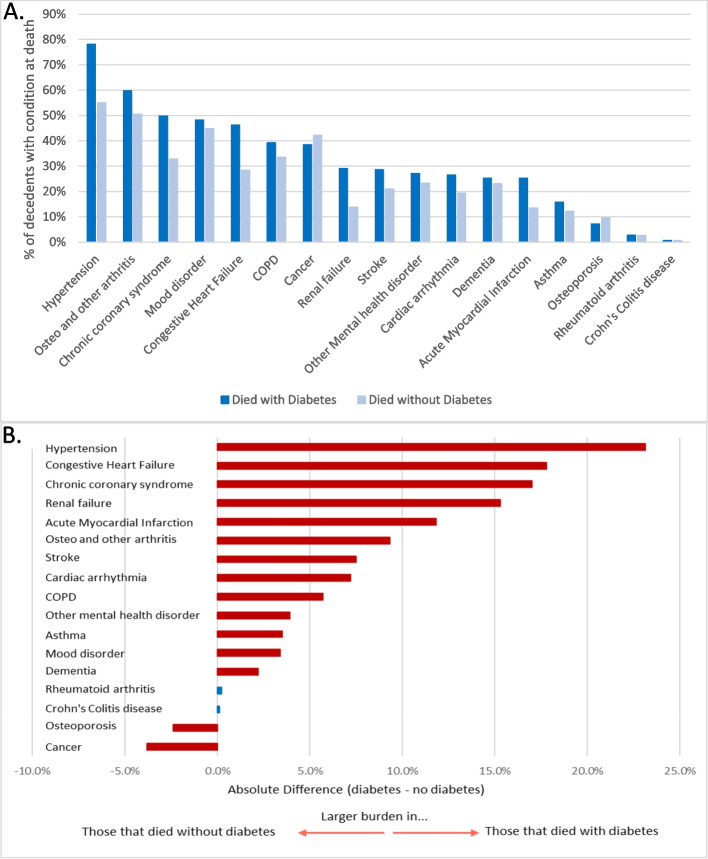
Chronic conditions at time of death by presence of diabetes, 1992–2017. **A** Percentage of adults that died with each chronic condition. **B** Absolute percentage differences in chronic conditions at time of death between decedents with and without diabetes

The distribution of chronic conditions at time of death is displayed in Fig. 1, panel A. The most prevalent chronic conditions at time of death for those that died with diabetes were hypertension (78.1%), osteo and other arthritis (59.8%), chronic coronary syndrome (49.8%), mood disorder (48.2%), and congestive heart failure (46.2%). Among those that died without diabetes, the top prevalent conditions at time of death were hypertension (55%), osteo and other arthritis (50.5%), mood disorder (44.8%), cancer (42.2%), and chronic coronary syndrome (32.8%). The absolute differences depicted in Fig. 1, panel B indicate a larger burden in most chronic conditions at time of death among those that died with diabetes. The largest absolute differences were observed for hypertension (23.1%), congestive heart failure (17.8%), chronic coronary syndrome (17.0%), renal failure (15.3%), and acute myocardial infarction (11.8%). In contrast, larger burdens in those that died without diabetes were found for cancer and osteoporosis, however, at smaller magnitudes for both (3.8% and 2.4%, respectively). Crohn’s colitis disease and rheumatoid arthritis at time of death had absolute differences of nearly 0, indicating negligible differences across the subsets.

### Causes of death

**Fig. 2:**
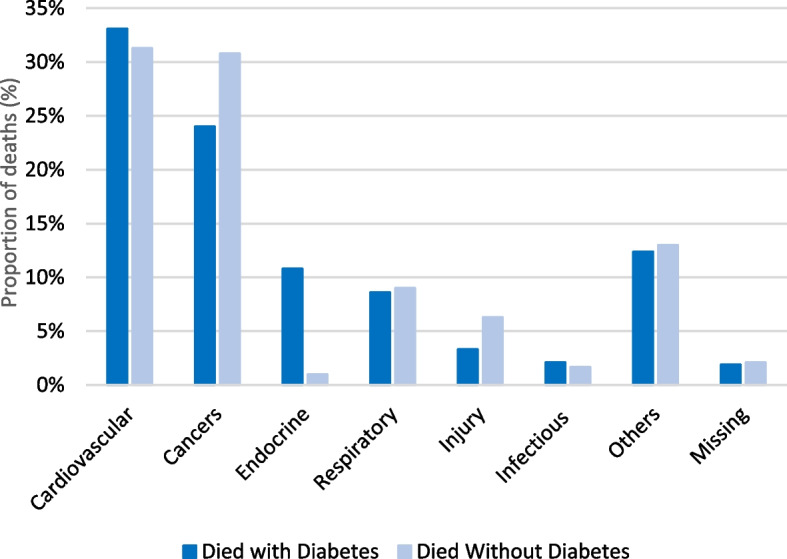
Cause of death by presence of diabetes, 1992–2017

Figure 2 displays the distribution of the causes of death among the cohort. The most common cause of death for both those that died with and without diabetes was cardiovascular and circulatory causes, however, this was slightly higher among those that died with diabetes than those without (33.1% compared to 31.3%). The largest difference between the groups occurred for endocrine causes, with the larger burden among those that died with diabetes (10.8% compared to 1.0%). Causes of death from cancer was more prevalent among those that died without diabetes (30.8% compared to 24.0%) and was also the second leading cause of death in the cohort. All other causes of death (respiratory, injury, infectious diseases, and others) were present at mid-low proportions (less than 15%) and were more common among those that died without diabetes, except for infectious causes.

### Effect of income and immigrant status on the presence of diabetes at time of death

Results from the log-binomial model show that relative to long-term residents, immigrants had a 19% higher risk (risk ratio (RR) 1.19, 95% CI: 1.18–1.20) and refugees had a 15% higher risk (RR 1.15, 95% CI: 1.12–1.18) of having a diabetes diagnosis at the time of death, after adjusting for age, sex, calendar year of death, income, and ADG score (see Table 2). An income gradient was also observed in that individuals who resided in the lowest neighbourhood income quintile at time of death had a 19% (RR 1.19, 95% CI: 1.18–1.20) higher risk of diabetes diagnosis compared to those in the highest income quintile, adjusting for other factors.

**Table 2:** Unadjusted and adjusted risk ratios (RR) for the presence of diabetes at the time of death (all-cause mortality), 1992-2017^a^

**Variables**	**Unadjusted RR (95% CI)**^**b**^	**Adjusted RR**^**a**^** (95% CI)**^**b**^
Male vs Female	1.13 (1.12, 1.13)	1.15 (1.14, 1.15)
Age	1.01 (1.01, 1.01)	1.00 (1.00, 1.00)
Immigrant vs Long Term Resident	1.26 (1.25, 1.27)	1.19 (1.18, 1.20)
Refugee vs Long Term Resident	1.19 (1.16, 1.23)	1.15 (1.12, 1.18)
Income quintile 1 vs 5	1.19 (1.18, 1.20)	1.19 (1.18, 1.20)
Income quintile 2 vs 5	1.15 (1.14, 1.16)	1.15 (1.14, 1.16)
Income quintile 3 vs 5	1.12 (1.11, 1.12)	1.12 (1.11, 1.13)
Income quintile 4 vs 5	1.08 (1.07, 1.09)	1.08 (1.07, 1.09)
ADG	1.06 (1.06, 1.06)	1.05 (1.05, 1.05)

^a^Risk ratios of adjusted model are adjusted for calendar year of death, age at time of death, sex, immigrant status, income quintile, and ADG score. Risk ratios were calculated with log binomial model

^b^All risk ratios have a *p* value < 0.0001

## Conclusion

In this population-based study we used linked vital statistics, census, and administrative databases to examine causes of death, diagnoses of diabetes and other chronic conditions at time of death, and demographic characteristics among deaths recorded in Ontario, Canada. Decedents with diabetes generally had larger burdens of chronic conditions at the time of death than those that died without diabetes. There were also significant variations in the proportion of decedents with a history of diabetes across immigrant status and area income quintiles, highlighting potential areas to focus medical and public health intervention.

In this cohort, the leading cause of death in individuals who died with diabetes was cardiovascular diseases. The burden of cardiovascular diseases among individuals with diabetes was corroborated when examining the distribution of chronic conditions at the time of death in that we observed larger proportions in the presence of congestive heart failure, chronic coronary syndrome, acute myocardial infarction, stroke and cardiac arrythmia compared to those that died without diabetes. This is consistent with other research that has demonstrated an increased risk of cardiovascular disease related death among those with diabetes [30, 31]. Cancer was the second leading cause of death in the cohort, however, we observed lower proportions in the presence of cancer among those that died with a diabetes diagnosis compared to those without. Previous research has shown no significant differences in the prevalence of cancer among those with diabetes and the general population [5]. Our findings add to existing research by evidencing that over the life course cancers are shown to accumulate in lower proportions among those with diabetes. Future work should explore whether cause of death proportions in Ontario have changed over time, as has been demonstrated in the United States and Europe [32–36].

Among those with diabetes at the time of death, we observed higher proportions in the presence of conditions that are considered complications of diabetes (i.e., renal failure), but also higher proportions of mood disorders, other mental health disorders, osteo and other arthritis, asthma, and COPD, demonstrating that individuals with diabetes experience a higher comorbidity burden over their life course than those without diabetes.

In this study, those with diabetes were less likely to die prematurely, a result that appears to contradict the more pronounced mortality risks documented for those with diabetes in younger age groups [31, 37, 38]. Similar results, however, have been found in a study conducted in Bulgaria where those with diabetes aged < 70 had a higher survival probability than those without [39]. We found that those with diabetes in our cohort were more likely to die from a cause considered amenable to the health system, compared to those that died without diabetes, suggesting a need for improvements in public health and medical care interventions for those with diabetes.

Compared to long-term residents, immigrants and refugees were more likely to die with diabetes. Immigrants have commonly been observed to be healthier than long-term residents in Canada, known as the healthy immigrant effect [12]. The results from our analysis suggests that the healthy immigrant effect observed in previous studies for all-cause mortality [40], varies according to diabetes status. It is known that the health of immigrants deteriorates with longer residence in the host country [12]. Differential effects in mortality and multimorbidity burden by the length of time since immigration is an area that can be explored in future research. We also observed a socioeconomic gradient for the model results indicating a greater risk in the proportion of decedents with diabetes among lower income quintiles compared to the highest. This coincides with well-established gradients found in the literature in regard to diabetes burden as well as risk of mortality among those of lower socioeconomic status [16, 41].

Several practice and policy directions have been proposed to respond to the growing multimorbidity burden, such as the implementation of integrated health care initiatives, chronic disease prevention, and efforts to address health inequities in multimorbidity [42, 43]. Our findings corroborate the need for such approaches and further demonstrate that policies and programming focused on reducing the multimorbidity burden warrant consideration of targeted efforts for the population with diabetes and sub-groups who are more at risk for developing diabetes.

A prominent strength of our study was the use of a large mortality database that captured all deaths registered in the province of Ontario over the study period, and the cohort was linked with administrative databases from a single-payer health system which enabled the identification of decedents history of chronic conditions at the time of death. As well, we identified all conditions for which there is evidence for the algorithm’s sensitivity and specificity in the administrative data.

The findings of this study should be interpreted in the context of several important limitations to the study methods. First, the study may have underestimated the multimorbidity burden given that administrative data was available until 1988, which resulted in a shorter look-back period for deaths that were registered in the earlier years. Second, our study relied on neighbourhood level income information based on postal code information at the time of death which is not a proxy for individual-level income [44]. Nonetheless, area-level income measures reflect important socioeconomic information about resident populations which can be informative for program and policy decision-making. Third, not all immigrants were identified in this analysis because the IRCC linkage starts in 1985 and does not include immigrants who initially landed in a province outside of Ontario.

Our findings add a novel understanding of the multimorbidity experience in those with diabetes from a life course perspective. We demonstrated that individuals who died with a diabetes diagnosis had a higher multimorbidity burden and were more likely to die from an amenable cause compared to those who died without diabetes. We also showed that immigrants, refugees and people of low socioeconomic status had a higher risk for the presence of diabetes at the time of death. The research suggests that efforts to reduce the multimorbidity burden will require inclusion of program and policy actions that are targeted for the population diagnosed with diabetes and policies and efforts to address the social determinants of health. This analysis underscores the role of health equity considerations in meeting the needs of population groups at risk for diabetes.

## Supplementary Information

**Additional file 1.** Definitions for Chronic Conditions.

**Additional file 2: Supplemental Table S2.** List of causes of deaths amenable to the Health System.

**Additional file 3.** Cohort Inclusion and Exclusion Criteria.

## Data Availability

The dataset used in this study is held securely in coded format at ICES. ICES is a prescribed entity under section 45 of Ontario’s Personal Health Information Protection Act. Section 45 authorizes ICES to collect personal health information, without consent, for the purpose of analysis or compiling statistical information with respect to the management of, evaluation or monitoring of, the allocation of resources to or planning for all or part of the health system. Legal restrictions and data sharing agreements prohibit ICES from making the dataset publicly available. Access may be granted to those who meet the conditions for confidential access, available at https://www.ices.on.ca/DAS.

## Abbreviations

RPDB: Registered Persons Database
ADG: Aggregated Diagnosis Groups (ADG)
DA: Dissemination Area
ODD: Ontario Diabetes Database
ORG-D: Office of the Registrar General- Deaths
ICD: International Classification of Disease
IRCC: Immigration, Refugees, and Citizenship Canada
ICES: In October 2018, the institute formerly known as the Institute for Clinical Evaluative Sciences formally adopted the initialism ICES as its official name
