# Supplementary material for: Multimorbidity at time of death among persons with type 2 diabetes: a population-based study in Ontario, Canada
Source: BMC Endocr Disord. 2023 Jun 2;23:127. doi: 10.1186/s12902-023-01362-x (PMC10236755; doi:10.1186/s12902-023-01362-x)
Supplement: Supplementary file 2 — Additional file 2: Supplemental Table S2. List of causes of deaths amenable to the Health System. [file 12902_2023_1362_MOESM2_ESM.docx]

| **Supplemental Table S2: List of causes of deaths amenable to the Health System** | | |
| --- | --- | --- |
| **Cause** | **ICD – 9 Code** | **Age group (years)^1^** |
| **Deaths amenable to both**  **medical care & public health**  Ischaemic heart disease | 410 – 414, 429.2 | 35 – 74 |
|  |  |  |
| **Deaths amenable to medical care** |  |  |
| Intestinal infection | 001 – 009 | 0 – 14 |
| Tuberculosis | 010 – 018, 137 | 0 – 74 |
| Diphtheria | 032 | 0 – 74 |
| Whooping cough | 033 | 0 – 14 |
| Tetanus | 037 | 0 – 74 |
| Septicemia | 038 | 0 – 74 |
| Poliomyelitis | 045 | 0 – 74 |
| Measles | 055 | 1 – 14 |
| Syphilis | 090 – 097 | 0 – 74 |
| Other bacterial infections | 019 – 031, 034, 320 – 322, 381 – 383, 390 –392, 680 – 686, 711 | 0 – 74 |
| Female breast cancer | 174 | 25 – 74 |
| Cervical cancer | 180 | 15 – 74 |
| Other uterine cancers | 179, 182 | 15 – 74 |
| Testicular cancer | 186 | 0 – 74 |
| Hodgkin’s disease | 201 | 0 – 74 |
| Leukemia | 204 – 208 | 0 – 14 |
| Diseases of the thyroid | 240 – 246 | 0 – 74 |
| Diabetes mellitus | 250 | 0 – 74 |
| Deficiency anemia | 280, 281 | 0 – 74 |
| Epilepsy | 345 | 0 – 74 |
| Active rheumatic fever | 390 – 392 | 0 – 74 |
| Chronic rheumatic heart disease | 393 – 398 | 0 – 74 |
| Hypertensive disease | 401 – 405 | 35 – 74 |
| Cerebrovascular disease | 430 – 438 | 35 – 74 |
| Influenza | 487 | 0 – 74 |
| Pneumonia | 480 – 483, 485 – 486 | 0 – 74 |
| Other acute respiratory infections | 460 – 466 | 1 – 14 |
| Asthma | 493 | 0 – 49 |
| Peptic ulcer | 531 – 534 | 0 – 74 |
| Appendicitis | 540 – 543 | 0 – 74 |
| Abdominal hernia | 550 – 553 | 0 – 74 |
| Ileus without hernia | 560 | 0 – 74 |
| Cholelithiasis, cholecystitis and cholangitis | 574 – 575.1, 576.1 | 0 – 74 |
| Nephritis and nephrosis | 580 – 589 | 0 – 74 |
| Infections of the urinary system | 590, 595 | 0 – 74 |
| Hyperplasia of the prostate | 600 | 0 – 74 |
| Complications of pregnancy | 630 – 676 | 0 – 74 |
| Osteomyelitis and periostitis | 730 | 0 – 74 |
| Congenital cardiovascular anomalies | 745 – 747 | 0 – 74 |
| Congenital digestive anomalies | 750 – 751 | 0 – 74 |
| Perinatal conditions, excluding still births* | 760 – 779 | 0 – 74 |
| Misadventures to patients during surgical and medical care | E870 – 876, E878 – 879 | 0 – 74 |
|  |  |  |
| **Deaths amenable to public health** |  |  |
| HIV | 042 | 0 – 74 |
| Lung cancer | 162 | 0 – 74 |
| Skin cancer | 173 | 0 – 74 |
| Chronic obstructive pulmonary disease | 490 – 492, 496 | 0 – 74 |
| Cirrhosis of the liver | 571 | 0 – 74 |
| Motor vehicle accidents | E810 – 825 | 0 – 74 |

**^1^**Age groups listed here are based on a standardized definition of amenable mortality (Nolte and McKee, BMJ 2003). In this study, all deaths occurred after age 20; as such, the true minimum age is 20 for all causes of death.
