# Supplementary material for: Multimorbidity at time of death among persons with type 2 diabetes: a population-based study in Ontario, Canada
Source: BMC Endocr Disord. 2023 Jun 2;23:127. doi: 10.1186/s12902-023-01362-x (PMC10236755; doi:10.1186/s12902-023-01362-x)
Supplement: Supplementary file 3 — Additional file 3. Cohort Inclusion and Exclusion Criteria. [file 12902_2023_1362_MOESM3_ESM.docx]

**Additional File 3: Cohort Inclusion and Exclusion Criteria**

**Age < 20 or ≥ 120**

N = 190

**Non-Ontario residents**

N = 561

**Death before diagnosis**

N = 46

**Age < 20 or ≥ 120**

N = 42 144

**Non-Ontario residents**

N = 4 882

**ORG-D and RPDB deaths linked to ODD**

N = 2 247 624

**Deaths in RPDB from 1 Jan 1992 – 31 Dec 2017**

N = 2 227 503

**Deaths in ORG-D from 1 Jan 1992 - 31 Dec 2017**

N = 2 198 177

**Included decedents without diabetes**

N = 1 552 960

**Included decedents with diabetes**

N = 646 841

**No diabetes diagnosis**

N = 1 599 986

**Diabetes diagnosis**

N = 647 638

**Total combined cohort**

N = 2 199 801

**Merged ORG-D and RPDB deaths**

N = 2 247 624
